# EYFP fusions to HD-Zip IV transcription factors enhance their stability and lead to phenotypic changes in *Arabidopsis*

**DOI:** 10.1080/15592324.2022.2119013

**Published:** 2022-09-26

**Authors:** Bibek Subedi, Kathrin Schrick

**Affiliations:** Division of Biology, Molecular, Cellular and Developmental Biology, Kansas State University, Manhattan, KS, USA

**Keywords:** EYFP, HD-Zip IV transcription factors, *Arabidopsis*, protein stability, abaxial curly leaf, GLABRA2

## Abstract

Green fluorescent protein (GFP) and its derivatives are extensively used for labeling cells, monitoring gene expression and/or tracking the localization or interactions of proteins. Previous reports of detrimental effects of fluorescent protein (FP) expression include cytotoxicity and interference with fusion protein function or localization. Only a few studies have documented the fluorescent tag-specific effects in plants. Here, we show that placing an enhanced yellow FP (EYFP) tag on the amino-terminus of GLABRA2 (GL2) and PROTODERMAL FACTOR2 (PDF2), two developmentally important HD-Zip IV transcription factors from *Arabidopsis*, enhances their protein stability. Additionally, expression of EYFP:GL2 not only rescued the *gl2* null mutant but also resulted in the abnormal development of abaxially curled leaves associated with EYFP-tag induced GL2 overexpression. Our study raises concerns on the use of FPs regarding their effects on the native properties of target proteins as well as biological consequences of fusion protein expression on morphology.

## Introduction

First observed in the jellyfish *Aequorea victoria* in 1962,^1,2^ green fluorescent protein (GFP) and its derivatives have revolutionized the molecular biosciences. Using fluorescent proteins (FPs) as tags, it is possible to visualize proteins and to mark organelles, intact cells or whole organisms. FPs are used extensively for analyzing gene expression, reporting promoter activity, monitoring protein subcellular localization, studying protein-protein interactions, protein purification, designing biosensors or cell markers, and lineage tracing.^3–6^ Their widespread popularity is based on fast and spontaneous formation of chromophores by posttranslational folding in the presence of oxygen without the need of any additional cofactors, substrates, or enzymes.^7^ FPs are generally compatible in fusion proteins, resistant to proteolytic enzymes and can be easily visualized with standard filters.^8,9^

Despite their above-mentioned useful properties, limitations of FPs have been increasingly recognized. GFP and its homologs are ~28 kDa proteins that form an eleven stranded beta-barrel structure with a length of ~4.2 nm and a diameter of ~2.4 nm.^1,10,11^ Addition of these relatively bulky tags can potentially interfere with protein folding and function as well as trafficking of proteins. However, limited evidence is available for such effects of FPs on fusion proteins. Some undesirable properties of FPs include their propensity to oligomerize, pH sensitivity, photobleaching, and overlap of their spectral signals with autofluorescence of cellular components.^12–17^ Their expression can also have deleterious effects on cells, as exemplified by the formation of cytotoxic aggregates,^6,12,18^ induction of apoptosis,^19,20^ and organ pathology in mice.^21,22^ One report showed that GFP expression in endothelial cells causes transcriptional upregulation of a heat shock protein, resulting in induction of cyclooxygenase-2, prostaglandin production and an increase in regional blood flow.^23^ In *Arabidopsis*, the presence of GFP at the amino- or carboxyl-terminus of a virus movement protein sterically hindered its ordered aggregation into tubules required for virus spread.^24^ Similarly, overexpression of GFP fused to the actin-binding domain of mouse talin (GFP:mTalin), which is typically used to study the actin cytoskeleton, attenuated blue-light-induced chloroplast movements in *Arabidopsis* leaves.^25^ In a separate study, overexpression of the same fusion protein caused a reduction in actin depolymerization, resulting in severe defects in root hair expansion.^26^

Here, we report the effects of enhanced yellow fluorescent protein (EYFP) tags on class IV homeodomain leucine-zipper (HD-Zip IV) transcription factors from *Arabidopsis*. These regulatory proteins are specific to epidermal or sub-epidermal cells of plants and play an important role in differentiation of these cell layers, anthocyanin accumulation, lipid transport, cuticle biosynthesis and responses to stress (^27–30^ reviewed in^31^). GLABRA2 (GL2), the founding member of this family from *Arabidopsis*, is expressed in developing trichomes and neighboring epidermal cells in leaves during early stages of tricome development.^32,33^ Later, GL2 is mainly expressed in trichomes and is required for the subsequent stages of their morphogenesis including cell expansion, branching and maturation of the trichome cell wall.^33^ GL2 is also preferentially expressed in the differentiating hairless cells in the root and hypocotyl epidermis^34–36^ and is involved in the inhibition of root hair initiation in non-hair cell files.^34,36,37^ It also regulates mucilage biosynthesis in the seed coat.^38,39^ Another HD-Zip IV member, PROTODERMAL FACTOR2 (PDF2), functions redundantly with *Arabidopsis thaliana* MERISTEM LAYER1 (ATML1) in shoot epidermal differentiation during embryonic development.^40,41^ Both genes positively regulate the expression of a subset of genes specific to the outermost layer (L1) of the shoot apical meristem.^28,40,42,43^

In numerous previous studies, FP tags were translationally fused to HD-Zip IV TFs to study their tissue-specific gene expression patterns, determine protein subcellular localization, and analyze mutant variants.^30, 44–48^ In particular, our previous work demonstrated that an EYFP-tagged GL2 protein rescues the null mutant phenotype of *gl2* plants, including rescue of defects in three different cell types: trichomes, non-hair root epidermal cells, and seed coat cells.^45,49^ However, tag-induced side effects have not been described to date. In this study, we report the effect of amino-terminal EYFP fusions on the stability of two HD-Zip IV transcription factors, GL2 and PDF2, along with an abnormal curly leaf phenotype associated with elevated expression of EYFP-tagged GL2.

## Results

### EYFP fusions to HD-Zip IV TFs enhance their protein stability

**Figure 1.**
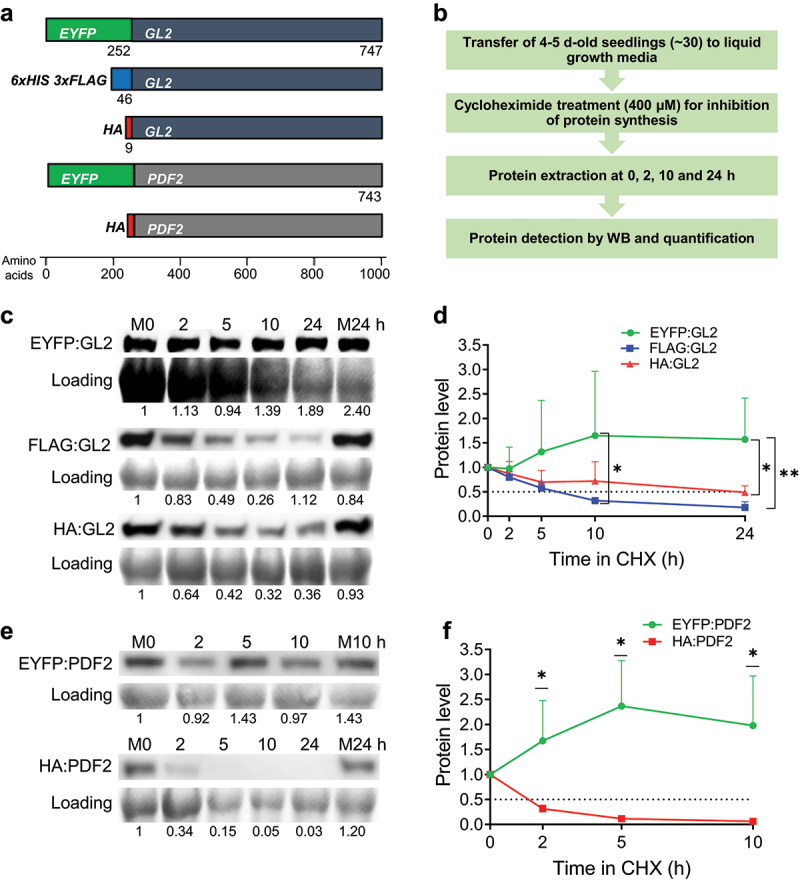
EYFP fusions to HD-Zip IV TFs enhance their protein stability. (a) Schematic of constructs (drawn to scale) used in this study including EYFP-, FLAG- and HA-tagged GL2 and EYFP- and HA-tagged PDF2. EYFP- and FLAG-tagged proteins were expressed under the *GL2* native promoter while HA-tagged proteins were expressed under the CaMV *35S* promoter. (b) Flow diagram of the in vivo CHX chase experiment used to monitor protein turnover. (c-f) Blot images and corresponding data quantification from CHX experiments for GL2 (c, d) and PDF2 (e, f) proteins. M0, M10 and M24, Mock treatments for 0, 10 and 24 h; Loading, RuBisCo bands from Coomassie Blue stained total protein. Means are shown for n = 3 biological replicates, and error bars signify standard deviations (SDs). Unpaired *t*-test or ordinary two-way ANOVA using Tukey’s multiple comparisons test was applied to test the significance of the difference between protein levels (*p < .05, **p < .005).

EYFP is a GFP variant comprising of S65G, V68L, S72A, and T203Y substitutions relative to wild-type GFP.^10,50^ Its excitation and emission peaks at 514 nm and 527 nm, respectively, are associated with stacking of Tyr66 with Tyr203.^10,51^ Here, we used transgenic *Arabidopsis* lines expressing amino-terminal fusions of EYFP to the HD-Zip IV transcription factors GL2 and PDF2 in comparison to amino-terminal fusions using 3xFLAG or HA tags (Figure 1a). The FLAG tag, also known as the DYKDDDDK-tag, is a short, hydrophilic epitope tag of ~1.013 kDa^52^ that is commonly implemented in a tandem configuration as the 3xFLAG peptide.^53^ Similarly, the HA tag is a ~ 1.102 kDa peptide corresponding to residues 98–106 of human influenza virus hemagglutinin (HA).^54^ For the EYFP and 3xFLAG tags, the fusion proteins were expressed under the *GL2* native promoter, whereas the cauliflower mosaic virus (CaMV) *35S* promoter was used to drive the HA-tag fusions.

To determine the effect of EYFP fusions on protein stability, we performed Western blot (WB) analysis of protein samples taken at various time points after treatment of *Arabidopsis* seedlings with cycloheximide (CHX) (Figure 1b). CHX is an inhibitor of eukaryotic translational elongation that is routinely used to study protein turnover in the absence of translation. Quantification of blot images showed that while the protein levels of GL2 fused to the smaller tags, 3xFLAG and HA, dropped over time with half-lives of ~6.5 and ~22 h respectively, EYFP:GL2 remained stable throughout the 24 h time course (Figures 1(c,d)). A more dramatic difference was observed between the half-lives of EYFP- and HA-tagged PDF2. The EYFP:PDF2 protein remained significantly more stable compared to HA:PDF2, which exhibited hardly detectable protein levels after 2 h (Figures 1(e,f)). These data indicate that the amino-terminal fusion to EYFP confers stability to HD-Zip IV TFs in plants.

### *Expression of EYFP:GL2 results in downward leaf curling in* Arabidopsis

**Figure 2.**
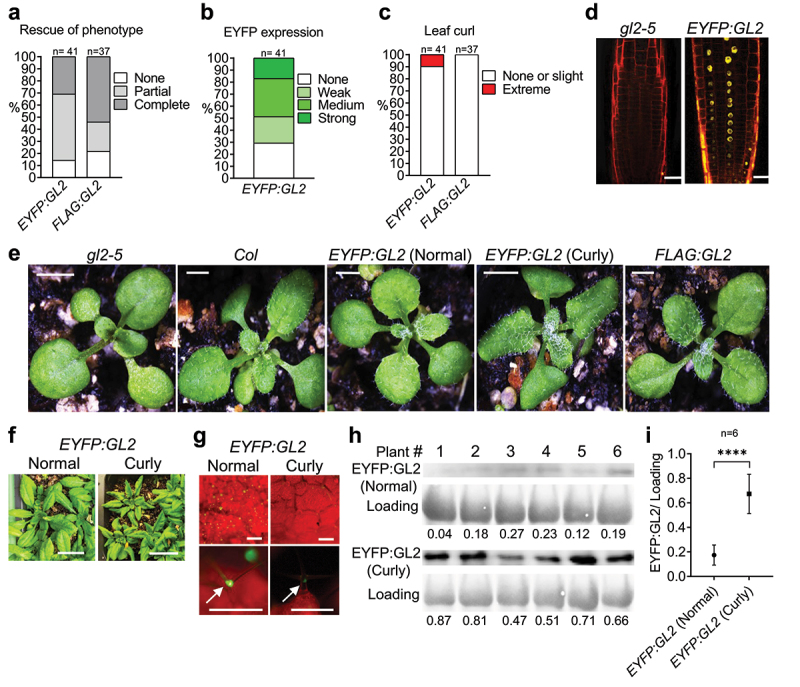
Expression of EYFP:GL2 leads to development of abaxially curled leaves in *Arabidopsis*. Graphs showing (a) rescue of trichome phenotype, (b) EYFP expression and (c) occurrence of curly leaves in T1 progeny of plants stably transformed with the indicated construct. (d) Confocal laser scanning microscopy indicating the nuclear localization of EYFP:GL2 in the primary root of a 4-d-old seedling stained with propidium iodide. Size bar = 20 μm. (e) Rosettes of 15-d-old wild type (Col), *gl2-5, EYFP:GL2* and *FLAG:GL2* plants. Size bar = 2 mm. (f) 25-d-old *EYFP:GL2* plants with two types of leaves. Size bar = 2 cm. (g) Expression of EYFP:GL2 in rosettes and leaf trichomes of 15-d-old plants. Size bar = 500 µm. (h) Blot images and (i) corresponding quantification data of EYFP:GL2 expression in the leaves of 25-d-old plants. Numerical values indicating EYFP:GL2 protein levels are represented as mean ± SD (n = 6 biological replicates). Unpaired *t*-test (two-tailed) was applied to test the significance of the difference between protein levels (****p < .0001).

Previous studies revealed the role of GL2 in the differentiation of leaf epidermal cells, especially in the formation of trichomes,^32,33^ but GL2 has not been implicated in leaf polarity establishment to date. In our study, screening T1 transformants for the rescue of null phenotypes indicated complete rescue of trichome defects in 30.95% (n = 42) and 54.05% (n = 37) of *EYFP:GL2* and *FLAG:GL2* transformants, respectively (Figure 2a). The *EYFP:GL2* lines exhibited a range of EYFP expression including strong (17.07%), medium (31.71%), weak (21.95%) and no (29.27%) expression (Figure 2b). Strikingly, 9.76% of *EYFP:GL2* plants displayed abnormal curling of leaves, a phenotype that was absent in *FLAG:GL2* lines (Figure 2c). Confocal laser scanning microscopy confirmed the nuclear localization of the EYFP:GL2 protein in the primary root of *Arabidopsis* seedlings (Figure 2d) indicating that fusing EYFP at the amino-terminus of GL2 does not alter its subcellular localization, consistent with previous reports.^45,49^ Taken together, our observations led us to hypothesize that the elevated protein stability of the EYFP:GL2 protein causes abnormal leaf curling during development.

**Figure 3.**
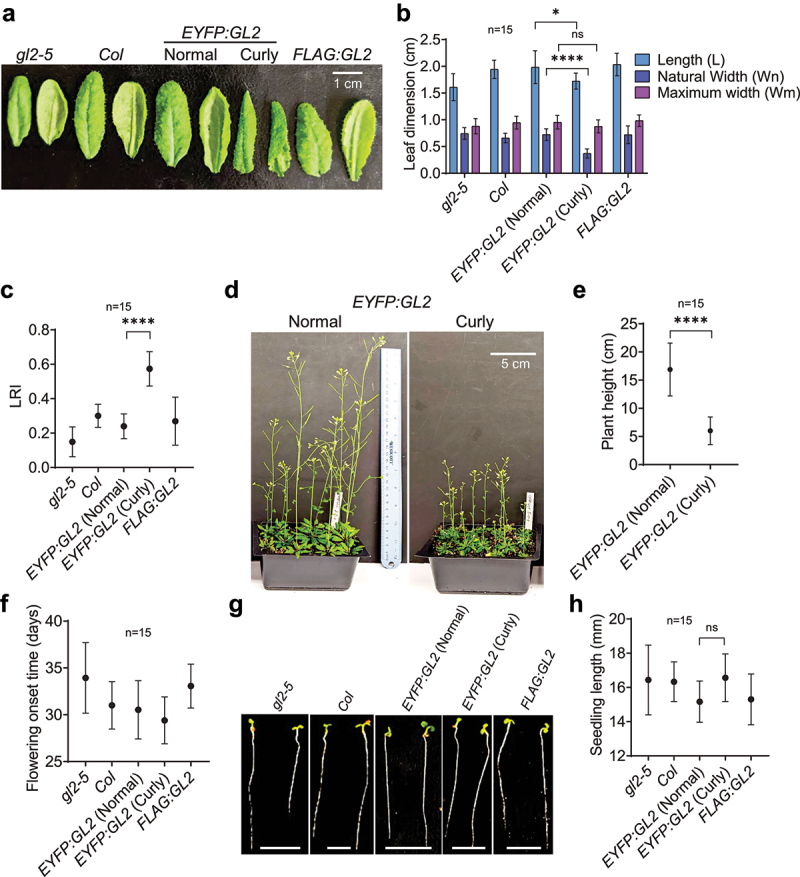
Quantitative characterization of curly leaf plants in comparison to normal/flat leaf plants. (a) The phenotypes of adaxial (left) and abaxial (right) faces, and (b) leaf blade length and width statistics of the 6^th^ leaves of 25-d-old plants. (c) Degree of leaf 6 epinasty in 25-d-old plants of various genotypes. (d) Images of 45-d-old *EYFP:GL2* plants with two types of leaves and (e) corresponding graph indicating their heights. (f) Time of onset of flowering in *EYFP:GL2* plants bearing normal and curly leaves. (g) Images of 4-d-old seedlings of various genotypes and (h) graph showing their lengths. Size bar = 5 mm. For all measurements, means are shown for n = 15 plants, and error bars indicate SDs. Depending upon the number of variables, ordinary one- or two-way ANOVA using Tukey’s multiple comparisons test was used to establish significant differences between genotypes (*p < .05, ****p < .0001).

The curly leaves in *EYFP:GL2* plants display a narrow and pointed morphology associated with downward rolling and altered adaxial-abaxial leaf polarity (Figures 2(e,f) and 3a). The leaf curling phenotype largely contributed to the distinct appearance of *EYFP:GL2* rosettes in comparison to *FLAG:GL2, gl2-5* null mutant or Col wild-type lines (Figure 2e). The onset of this phenotype occurred at around day 12–13 after germination, with rolling occurring in either the longitudinal or transverse direction (Figure 2e). However, longitudinal curling became predominant later in development and the intensity of leaf curling increased at the stages leading up to flowering (Figure 2f). We next asked whether the EYFP:GL2 protein expression level is altered in curly leaf plants in comparison to normal leaf plants. Under an epifluorescence stereomicroscope, fluorescent signals in leaf trichome nuclei of curly leaf plants appeared much stronger compared to those of normal leaf plants (Figure 2g). To confirm this, WB analysis was performed with leaves from 25-d-old curly leaf and normal leaf plants expressing EYFP:GL2 (Figure 2h). Quantification of protein bands revealed significantly higher expression of EYFP:GL2 in curly leaves compared to normal/flat leaves (Figure 2i).

### Characterization of the curly leaf trait associated with EYFP:GL2 expression

Leaf size and shape play important roles in overall plant architecture and physiology.^55,56,56^ Appropriate leaf rolling is of agronomic value as it is associated with erect leaf canopies, higher photosynthetic efficiency, optimum canopy temperature, and protection against UV light.^57–59^ Leaf rolling is also critical for adaptation to drought as it affects osmotic adjustment and reduces transpirational water loss.^60–62^ To quantify the effect of EYFP:GL2 expression on the three-dimensional architecture of leaves, we measured the distances between the leaf edges halfway from the leaf blades in curly leaf versus normal/flat plant lines. Measurement of the leaf dimensions revealed significantly reduced leaf blade width in curly leaf lines compared to the normal/flat leaf lines although both displayed similar leaf lengths (Figure 3(a,b)). In accordance, *EYFP:GL2* plants exhibited the highest values for the rolling leaf index (LRI) or degree of epinasty, followed by Col wild type, *FLAG:GL2* and *gl2-5* null mutant plants (Figure 3c). These quantitative data confirm our original observations that the leaf blade edges curl downward to a greater extent in *EYFP:GL2* curly leaf lines (Figures 2(d,e) and 3a). The curly leaf plants also exhibited short stature with somewhat stunted growth (Figures 3(d,e)) and slightly early flowering (Figure 3f) compared to plants with normal leaf morphology. In contrast, there were no significant differences in the lengths of the hypocotyl and root in young seedlings (Figures 3(g,h)).

## Discussion

In this communication, we quantified the stability of HD-Zip IV transcription factors fused to various tags and reported the phenotypic changes in *Arabidopsis* lines expressing highly stable EYFP:GL2 fusion proteins. There are several possible reasons behind the altered stability of fluorescently tagged HD-Zip IV proteins. Addition of the EYFP tag could potentially change protein conformation, interfere with binding partners or impact posttranslational modifications (PTMs) such as phosphorylation or ubiquitination, altering the extent of protein activation or degradation. In a related study using mammalian cells, expression of EGFP inhibited ubiquitination by the RING-type E3 ubiquitin ligases TRAF6 and API2, resulting in impaired NF-kB and JNK signaling and enhanced stability of the tumor suppressor p53.^63^ Intriguingly, it was found that EGFP contains a consensus motif (PNEKRD) implicated in TRAF6 binding.^63^ In another study, the expression of EGFP impaired actin-myosin interactions and thus contractile properties of cardiac muscle cells by competitive binding to the actin-binding site of myosin.^64,65^ Proteins also have targeting signals and sites for PTMs such as palmitoylation, myristylation and farnesylation^66–68^ which are reported to be obscured by fusion to fluorescent tags.^68,69^ Although the EYFP:GL2 protein is functional in terms of its ability to rescue *gl2* null mutants, amino-terminal tagging may nonetheless cause subtle changes in GL2 trafficking or turnover. Fusion to the EYFP tag could additionally disrupt another aspect of GL2 function when it is overexpressed, such as slightly altering its DNA-binding specificity, thus causing misexpression of target genes responsible for leaf curling.

In a previous study, it was reported that ectopic expression of *GL2* using the CaMV *35S* promoter disrupts the endogenous function of *GL2* and fails to rescue the *gl2-1* mutant.^70^ In the same study, additive expression of *GL2* under its native promoter in the wild-type genetic background resulted in an increase in trichome initiation along with altered spacing of trichomes.^70^ However, a curly leaf phenotype was not reported in either scenario that was associated with overexpression of untagged GL2. Therefore, the occurrence of leaf curl in our study seems to be attributed to the EYFP component of the fusion protein. Furthermore, the data presented here show that the more pronounced abaxial leaf curl or epinasty of leaf blade edges in a subset of *EYFP:GL2* lines was associated with the enhanced protein level and stability of GL2 (Figures 1(c,d), and 2(g–i)). Not all plants expressing EYFP:GL2 displayed this phenotype (Figures 2(c,e), 3(a–d)) because each transformation event represents a random insertion of the transgene into the genome and positions of the insertion and/or tandem insertions can impact expression levels (Figure 2b). Leaf curling is usually associated with polarity changes in the leaf abaxial–adaxial axis.^71,72^ A complex regulatory network consisting of various transcription factors including HD-Zip III,^73–76^ YABBY^77–79^ and KANADI (KAN),^80–84^ small non-coding/microRNAs^76,85,86^ and components of hormonal signaling including the *Auxin/Indole-3-Acetic Acid* (*Aux/IAA*) gene family and the redundantly acting *AUXIN RESPONSE FACTOR* (*ARF*) genes *ETTIN* (*ETT*)/*ARF3 ARF4*^57,87–89^ are known to control establishment of leaf polarity. Auxin biosynthesis, polar transport, and signal transduction are important for plant growth and development^90,91^ and defects in these processes can lead to leaf curling and dwarf/semi-dwarf phenotypes.^92–94^ Since we observed dwarf stature and reduced plant growth in association with leaf curling in plants expressing EYFP:GL2 (Figure 3(d,e)), it is possible that GL2 overexpression exerts its effect through auxin signaling or another hormone pathway.

A few HD-Zip IV genes were implicated in leaf curling previously. In one study, knockout of *Rice Outermost Cell-Specific 5* (*ROC5*), an ortholog of *GL2*, resulted in abaxial leaf rolling because of abnormal bulliform cell formation in the adaxial leaf blade.^95^ Vice versa, overexpression of *ROC5* suppressed the development of bulliform cells giving rise to adaxially rolled leaves in rice.^95^ However, we observed the opposite effect with GL2 overexpression in *Arabidopsis* (Figures 2(c,e,f), 3(a–d)). A more recent study showed that HD-Zip IV member ROC8 heterodimerizes with ROC5 in transcriptional repression of *Abaxially Curled Leaf 1* (*ACL1*), a positive regulator of bulliform cell development.^96^ Overexpression of GL2 seems to induce leaf curling by a different mechanism since bulliform cells are specific to monocots. One approach to identify the leaf polarity determinants affected by EYFP:GL2 expression might be transcriptomic analysis of differentially expressed genes in curly versus normal/flat leaves. Similarly, mRNA in situ hybridization could be used to identify tissue-specific or polarized distribution of specific transcripts that regulate leaf polarity.^89,97,98^ Since changes in epidermal cell shape, size or arrangement may be associated with leaf curling,^79^ anatomical examination of leaves with curly leaf morphologies will be useful in future studies.

Although the use of fluorescent tags is critical for specific experiments, it is often helpful to use alternative tags of different sizes and/or properties to rule out any tag-specific observations. If feasible, using specific antibodies against the proteins of interest will also aid in resolving tag-related issues, e.g., to determine whether the fusion proteins are overexpressed relative to their endogenous counterparts. The position of the tag may also influence protein activity. While our analysis was restricted to amino-terminal fusions, it would be interesting to see whether attaching EYFP to the carboxyl-terminus or inserting it internally between domains affects GL2 function and/or leaf curling. Nonetheless, chances of affecting protein structural features or disrupting interactions with a ligand or other proteins are high if the site of insertion is not selected properly. In summary, being aware of the limitations of fluorescent tags and available alternatives is important when generating fusion proteins in research studies.

## Materials and methods

### Plant materials and growth conditions

The wild-type (WT) *Arabidopsis thaliana* ecotype Columbia (Col), *gl2-5* null mutant^99^ and transgenic plants expressing EYFP or FLAG-tagged GL2 were grown on soil containing Berger BM6, vermiculite and perlite (Hummert International) in a ratio of 4:2:1 at 23°C under continuous light.

### Generation of transgenic lines

The *proGL2:EYFP:GL2* construct was generated using *SR54* binary vector as previously described.^45^ For FLAG tagging, the *proGL2:EYFP:GL2* construct was restriction digested with *BamH*I and *Sal*I to remove the EYFP tag and the 6xHis 3xFLAG tag from pB7HFC-GFP^100^ was added to amino-terminus of *GL2* using NEBuilder HiFi DNA Assembly (New England Biolabs, E5520S). Construction of *HA:GL2, EYFP:PDF2* and *HA:PDF2* is described elsewhere.^101^ The binary vector constructs were used to transform *gl2-5* plants by *Agrobacterium tumefaciens* (GV3101) mediated floral dip.^102^ Transgenic lines were screened in presence of 20 μg/ml Hygromycin B. For each genotype, 37–42 independent T1 transformants were characterized for rescue of the trichome phenotype, EYFP expression and leaf curling. T2 progeny were analyzed for 3:1 segregation of the transgene. T3 homozygous lines confirmed by PCR genotyping and sequencing were used for phenotypic analysis and further experiments.

### In vivo protein stability assay

Seeds from EYFP-, FLAG- and HA-tagged GL2 or PDF2 lines were vapor sterilized, sown on 0.5X MS agar,^103^ stratified at 4°C for 4–5 days, and germinated in vertical orientation at 23°C under continuous light. After 4 days, samples of ~30 seedlings each were transferred to 24-well plates containing 1 ml 0.5X liquid MS media, followed by the incubation on a shaker at 60 rpm for ~16 h under continuous light at 23°C. Next, cycloheximide (Sigma-Aldrich, C1988) (400 µM in DMSO) or DMSO control was added to the wells. Seedling samples were harvested in screw-cap microcentrifuge tubes at the designated times by immediate freezing in liquid nitrogen followed by storage at −80°C until further use. Proteins were extracted from the seedlings by adding Laemmli sample buffer at 95°C after grinding in liquid nitrogen. For Western blotting, samples were separated on Bio-Rad Mini-PROTEAN TGX gels (10%), transferred to PVDF membranes and incubated with primary antibodies against GFP (1:2000; Roche, 11814460001), FLAG (1:10,000; Sigma, F1804) or HA (1:10,000; Pierce, 26183) followed by goat anti-mouse secondary IgG HRP antibodies (1:3300; GenScript, A00160). Proteins were detected with SuperSignal West Femto Maximum Sensitivity Substrate (ThermoFisher Scientific) using an Azure 300 chemiluminescence imager (Azure Biosystems). The membranes were stained with Bio-Safe Coomassie Blue G-250 (Bio-Rad) for loading controls, and band intensities were quantified with ImageJ software.

### Phenotypic assays, leaf biometry, and microscopy

Rosettes and trichomes of ~15-d-old plants and EYFP expression were imaged with a Leica M165 fluorescence stereomicroscope fitted with a GFP2 filter set and a Leica DFC295 digital camera. To measure seedling lengths, sterile seeds were sown on 0.8% agar media, stratified at 4°C for 5 days and the plates were placed vertically at 23°C under continuous light for 4 days. The leaf blade lengths and widths were measured on the 6th leaves^104^ of 25-d-old plants of each genotype. Digital images of seedlings, whole plants and leaves were captured using a Google Pixel 3XL (12MP f/1.8) camera. To determine the subcellular localization of EYFP-tagged proteins, the primary roots of 4-d-old seedlings stained with propidium iodide (10 μg/ml) were imaged with a Zeiss LSM 880 confocal microscope. For acquiring EYFP signals, a 488 nm Argon laser and a 500–550 nm emission filter was used. For propidium iodide, a 561 nm DPSS laser in combination with a 575–645 nm emission filter was used.

Leaf Rolling Index (LRIs)/ Degree of epinasty was calculated following the protocol described in^105^ and.^57^

LRI = (Wm − Wn)/Wm × 100, where Wm is the greatest width of the leaf blade in its expanded state and Wn is the natural distance of the leaf blade margins at the same site.

Interpretation of LRI values:

0 = Flat leaf blade (no curling)

+ve = Epinastic leaf blade (downward curling)

-ve = Hyponastic leaf blade (upcurling)
